# Robust Population Inversion by Polarization Selective Pulsed Excitation

**DOI:** 10.1038/srep10313

**Published:** 2015-05-22

**Authors:** D. Mantei, J. Förstner, S. Gordon, Y. A. Leier, A. K. Rai, D. Reuter, A. D. Wieck, A. Zrenner

**Affiliations:** 1 ^1^Physics Department and Center for Optoelectronics and Photonics Paderborn (CeOPP), Universität Paderborn, Warburger Straße 100, 33098 Paderborn, Germany; 2 Theoretical Electrical Engineering and Center for Optoelectronics and Photonics Paderborn (CeOPP), Universität Paderborn, Warburger Straße 100, 33098 Paderborn, Germany; 3 Ruhr-Universität Bochum, Universitätsstraße 150, Gebäude NB, 44780 Bochum, Germany

## Abstract

The coherent state preparation and control of single quantum systems is an important prerequisite for the implementation of functional quantum devices. Prominent examples for such systems are semiconductor quantum dots, which exhibit a fine structure split single exciton state and a V-type three level structure, given by a common ground state and two distinguishable and separately excitable transitions. In this work we introduce a novel concept for the preparation of a robust inversion by the sequential excitation in a V-type system via distinguishable paths.

The coherent manipulation and inversion of quantum systems based on Rabi rotations^1^,^2^ or the Adiabatic Rapid Passage^3^,^4^,^5^ are important foundations for the realisation of quantum gates^6^ and promising excitation schemes for deterministic single photon sources^7^,^8^. The achievements in this important field of quantum technologies are based on the coherent physics of two level systems^9^. In many cases however, the used quantum systems exhibit a V-type three level structure. A prototype for such a system is the fine structure split single exciton state in semiconductor quantum dots (QDs)^10^,^11^,^12^,^13^,^14^,^15^,^16^, which has a strong impact on the generation of polarization entangled photons via the sequential biexciton decay^17^,^18^,^19^. In semiconductor QDs V-type three level systems are caused by electron hole exchange interaction or by Zeeman interaction.

In QDs with anisotropic confinement potential, the electron hole exchange interaction leads to a fine structure splitting (FSS) *ΔE*_*FSS*_ of the single exciton state in a |X> and |Y> component^10^ (see Fig. 1). The two fine structure transitions, denoted as π_x_ and π_y_ are linearly and orthogonally polarized, in (001)-oriented zincblende materials typically along the 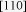 and 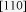 crystal axis. In the current contribution we focus on the ground state transition and we specifically take advantage of the FSS and the two orthogonally polarized transitions, which are distinguishable and separately excitable. We drive those transitions by cross-polarized double pulse excitation with resonant ps laser fields, which do not overlap in time. In a single quantum object the maximum occupancy remains however limited to one exciton due to Pauli blocking^20^. Here we show that the use of two independent excitation channels will create inversion conditions, which are robust against variations in the pulse areas. For the basic experimental demonstration of this approach we use a single InGaAs QD in a n-i-Schottky diode with a semi-transparent Schottky gate, which allows for electric Stark effect tuning of the energy levels and a measurement of the population by photocurrent spectroscopy^21^.

## Results

**Figure 2:**
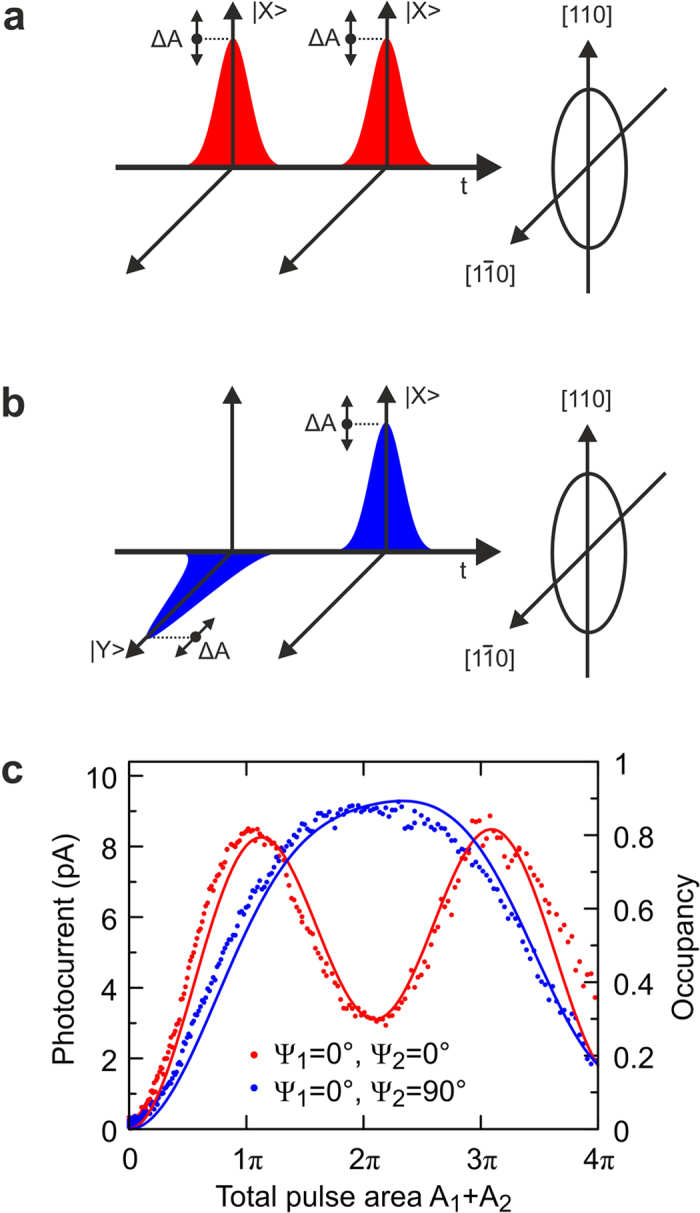
Schematic representation of double pulse excitation using co-polarized laser pulses with constructive phasing (**a**) and cross-polarized pulses (**b**). The pulse areas for the consecutive pulses are equal. Raising the total pulse area of both pulses (**c**) leads for the co-polarized case to Rabi oscillations (experiment: red dots; theory: red solid line). Sufficient degrees of inversion can only be obtained in narrow regions around 1*π* and 3*π*. Cross-polarized excitation (shown in blue) offers robust inversion with a flat gradient for a considerably wider range of pulse areas around 2*π*.

**Figure 3:**
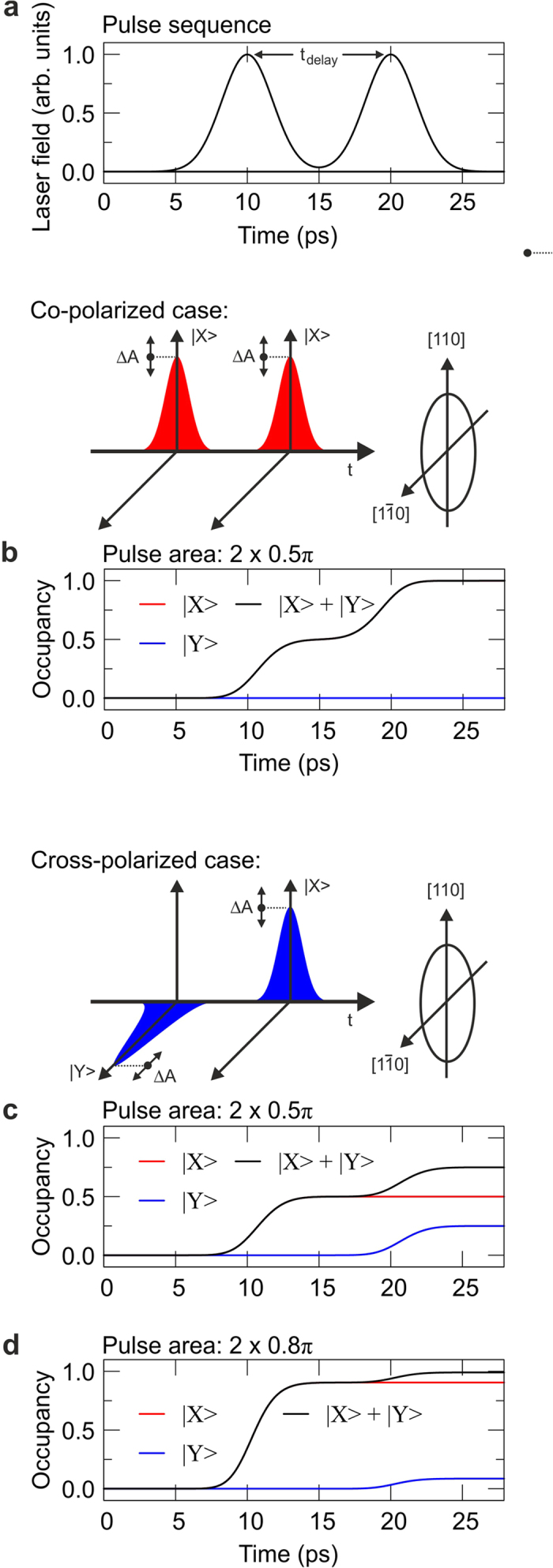
Calculated time evolution of the FSS states for the double pulse laser excitation (constructive phasing) shown in (**a**). |X> and |Y> occupancies versus time for the co-polarized case and 2 × 0.5 *π* pulse area (**b**) and for the cross-polarized case with 2 × 0.5 *π* (**c**) and 2 × 0.8 *π* pulse area (**d**).

In the following we denote the horizontally (vertically) polarized laser fields, which can only excite the |X> (|Y>) state, with the angle 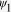
=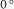
(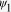
=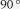). First, we discuss double pulse excitation with two horizontally polarized ps pulses (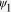
=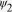
=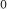) with equal pulse area (see Figs. 2a and 3a) and constructive phasing. For this special case the system is reduced to a two level system. An increase of the total pulse area (A_1_+A_2_) leads to the well-known Rabi oscillations in the total occupancy of the exciton^21^, as shown in Fig. 2c (theory: red solid line, experiment: red dots). The theoretical results shown in this work are solutions of the optical Bloch equations for a V-type three level system^22^. The theoretical data shown in Fig. 2c was calculated for the experimentally determined dephasing time T_2_ = 85 ps. At a total pulse area of *π* and 3*π* and in the absence of dephasing, each pulse generates an occupancy of 0.5, which is summed up to a complete population inversion of the system, if the time delay between the pulses is fine-tuned to constructive phasing. Figure 3b shows the simulated time development of the total occupancy for this case, which appears only in |X>.

**Figure 4:**
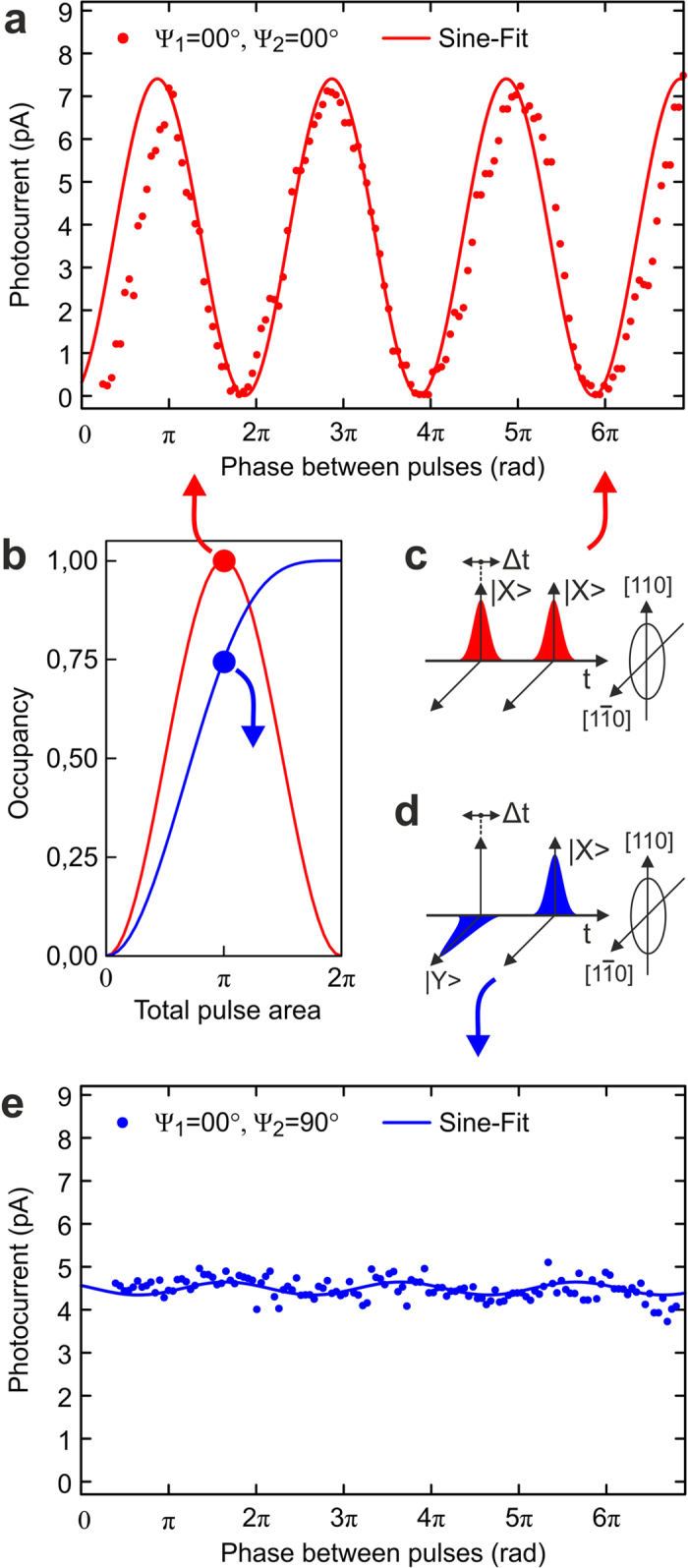
Ramsey interference experiments for co- and cross-polarized double pulse excitation with a total pulse area of *π*. (**a**) Co-polarized case as sketched in (**b**) and (**c**) Fine tuning of the phase leads to a full modulation of the occupancy, which is measured by photocurrent detection. (**e**) The phase-dependence of the occupancy virtually vanishes for cross-polarized excitation as sketched in (**b**) and (**d**).

Shifting the phase between both laser pulses (by varying the time delay) leads to Ramsey interference^23^, as shown in Fig. 4a for a total pulse area of *π* and co-polarized excitation (see red curves in Figs. 4b,c). For manipulation protocols, which require two or more pulses, the obligatory phase control remains here a big experimental challenge. Furthermore, small variations from *π* in the pulse area (or in the oscillator strength of the quantum system) lead to significant (quadratic) reductions of the total occupancy.

In contrast to the co-polarized scenario discussed above, the use of cross-polarized pulses (see Fig. 2b) shows a completely different behaviour in regard to population oscillations, as we show in Fig. 2c. Blue dots represent the experimental results and the blue solid line the corresponding theoretical data for the experimentally determined dephasing time T_2_ = 85 ps. In contrast to the co-polarized case, the maximum occupancy is reached for a total pulse area of 2*π*, with a very extended and flat gradient around this point. Variations in the total pulse area around 2*π* only lead to a small reduction of occupancy. This unique feature can also be seen very clearly in Fig. 4b, where theoretical results for vanishing dephasing are shown for both the co-polarized (red) and cross-polarized case (blue). The applied excitation scheme with cross-polarized pulses allows therefore for the robust and fault tolerant inversion of this V-type three level system.

The resulting occupancy is now made up of the sum of both fine structure states. Figure 3c shows the simulated time development of the FSS states for a total pulse area of *π*. The first horizontally polarized pulse excites the |X> FSS state to an occupancy of 0.5, the second vertically polarized pulse can only excite the |Y> state, leaving |X> unchanged. At this point it is already evident that the partial range in occupancy, which still can be manipulated in terms of Rabi rotations via the action of vertically polarized pulses on the |Y> state, remains limited to the interval between 0.5 and 1. Although the second pulse is a *π/*2 pulse too, it cannot excite the |Y> state from 0.0 to 0.5, but only up to 0.25, which results in a total occupancy of 0.50 + 0.25 = 0.75. This limitation is adequately confirmed by the experimental results shown in Fig. 2c (blue dots), where the photocurrent for a total pulse area of *π* (6.2 pA, at 80 MHz pulse repetition rate) is found to be 69% of the photocurrent obtained for 2*π* (9 pA).

The sequential generation of occupancy in the FSS basis also provides the explanation for the very flat gradient around the total pulse area of 2*π*. For example, in an excitation scenario with 0.8*π* pulses (instead of ideal *π* pulses) the first pulse makes a contribution of 0.904 to the total occupancy, whereas the second pulse lifts the occupancy additionally by 0.086 to a total occupancy of 0.99, as shown by the theoretical results in Fig. 3d. A 20% deviation from the ideal pulse areas leads therefore only to a 1% reduction of the resulting occupancy. So, for cross-polarized excitation, even imperfect pulse areas lead to a practically complete inversion of the V-type three level system.

Double pulse excitation with cross-polarized pulses, as sketched in Figs. 4b,d, also offers the advantage of phase independence between the pulses as shown in Fig. 4e. As already described above, the first horizontally polarized pulse creates a coherent superposition between |X> and |0>, leaving |Y> unaffected. The second vertically polarized pulse selectively adds a coherent superposition between |Y> and |0>, which is orthogonal to the first one and hence also insensitive to the phase of the first manipulation. Only the total occupancy as the sum of |X> and |Y> remains limited to 1 (exciton) due to Pauli blocking. This property of a V-type three level system offers therefore also the ability to realize an optical switch by controlling the absorption of the three level system in regard to a specific polarization. If, for instance, a first horizontally polarized *π* pulse already generated a complete inversion in the |X> state, a second vertically polarized pulse cannot excite the system any more regardless of its pulse area and phase and will hence be fully transmitted.

**Figure 5:**
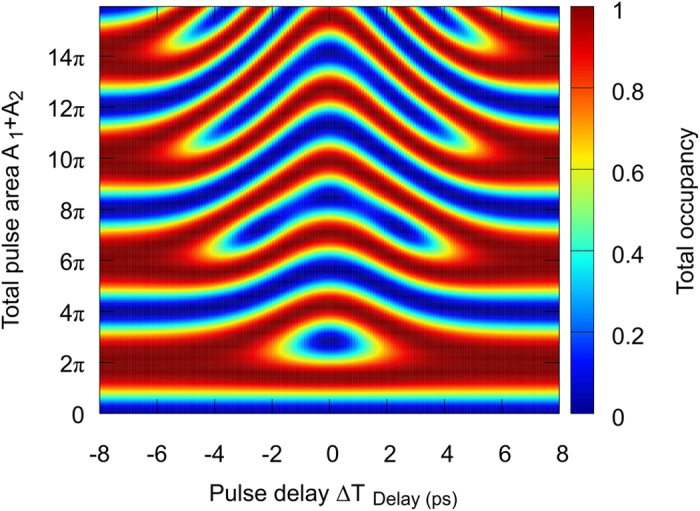
Calculated total occupancy (sum of |X> and |Y> states, colour coded) as a function of the pulse delay and the total pulse area for cross-polarized pulses (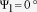, 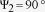). For zero pulse delay, the pulse area dependence corresponds to an ordinary Rabi scenario, as obtained for a single +45^*°*^ polarized pulse. Increasing or decreasing pulse delay results in a seamless transition to the discussed scenario with cross-polarized double pulses, which generates broad regions of robust inversion for total pulse areas at integer multiples of 2π.

To verify that our findings are consistent with the well-known Rabi scenario for co-polarized pulses, we have performed a simulation (see Fig. 5), which shows the resulting occupancy (|X> + |Y>) for cross-polarized pulses (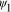
=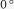, 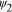
=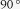) as a function of the pulse delay time 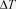 and the sum of the equal pulse areas A_1_+A_2_. For zero pulse delay (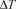
=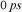) the pulses overlap in the time domain. The vector addition of both electromagnetic field components results in a single +45^°^ polarized pulse. A cut through the colour coded map at zero pulse delay shows therefore regular Rabi oscillations with increasing pulse area. The differences to the previously discussed co-planar case are the +45^°^ orientation of the polarization and the x-axis scaling for the total pulse area, leading to a Rabi oscillation period of 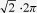 and a maximum occupancy at 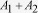
=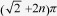 (with n 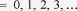). Increasing the pulse delay (regardless of the temporal order of the pulses) towards the regime of temporally fully separated pulses (at |Δ*T*|>6*ps*), leads to a shift and pair-wise fusion of Rabi oscillation maxima into broad regions 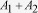
=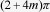
(with 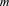
=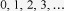). Fig. 5 shows this smooth transition over the entire regime from the 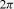-periodic Rabi-scenario into the 4Π‐ periodic regions of robust inversion. The appearance of robust inversion for the case of vanishing pulse overlap is controlled by the build-up of population in the |X> and |Y> components of the single exciton ground state, which is subjected to Pauli blocking as a whole.

## Conclusions

In summary, we have introduced a novel scheme for the generation of a robust inversion in a V-shaped three level system. An experimental demonstration was performed on the fine structure split single exciton transition of a single QD by resonant excitation with cross-polarized laser pulse pairs. Outstanding features of this excitation scheme are the great robustness against pulse area variations and the insensitivity with respect to phase relations between the excitation pulses. This population driven scheme, which gains its intrinsic nonlinearity from Pauli blocking in a single quantum system, allows also for the control of the absorption of a selected FSS transition by orthogonally polarized *π*-pulses.

## Methods

### Experiment

#### Used material system / Properties of the sample

We used a single InGaAs QD, which was grown employing the Stranski-Krastanov method by molecular beam epitaxy. It was embedded in a 310 nm thick intrinsic layer of a n-i-Schottky diode. The QD layer was 40 nm above the n-doped layer. The Schottky-contact was formed by a semi-transparent titanium layer to facilitate optical access to the quantum dot. Micron-sized etched apertures in an aluminium top layer where used as masks to laterally select a single QD out of a low density ensemble.

#### Photocurrent spectroscopy

A reverse bias voltage *V*_*B*_ was applied to tune the exciton ground state energy by use of the Quantum-Confined Stark effect (QCSE). With increasing *V*_*B*_ the tunnelling rate of electrons (and holes) out of the QD gets enhanced and reaches the spontaneous radiative recombination rate at about *V*_*B*_ = 0.25 *V*, which corresponds to about 30 kV/cm internal electric field. At even higher *V*_*B*_ the excitation in the QD can be extracted by photocurrent measurement (according to a rate equation with two loss terms). As outlined in reference^21^ and^23^, about 95% of the excitation can be extracted via photocurrent at sufficiently high V_B_. Also in the regime of incomplete tunnelling at lower *V*_*B*_, where the extractable photocurrent is below the theoretical limit of *I* = *f*·*e* (*f*: ps laser repetition frequency, *e*: elementary charge)^21^, the current, which corresponds to full inversion, can be determined^23^, allowing for a quantitative measurement of occupation also in this regime. For the data shown in Fig. 2c, occupancy 1 corresponds to a photocurrent of 10.4 pA.

#### Setup: Laser

For resonant excitation we used a mode locked Ti:sapphire laser with a pulse duration of 2.5 ps and a repetition rate of *f* = 80 MHz, which was tuned to resonance with the exciton FSS lines at *E* = 1.3612 *eV* (*λ* = 910.84 nm). The Fourier-transform-limited pulses were spectrally broader than the measured FSS 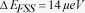, but narrow enough to excite nothing else than the one exciton transitions from |0> to |X> and |Y>. With this conditions it was ensured, that further transitions to the biexciton state did not occur.

#### Setup: Interferometer and delay

A Mach-Zehnder interferometer was used to generate the required pulse pairs. Each path of the interferometer was equipped with a half- and quarter-waveplate to prepare the polarization of each pulse independently. The polarization control was calibrated by use of a commercial free-space polarimeter. Therefore it was possible to compensate small distortions of the polarization, which were introduced by further optical components such as beamsplitters. Time delay and phase between the pulses where realized by an optical delay line in one of the interferometer paths, which consisted of a nanopositioner stage on top of a translation stage. The time delay was set to Δ *T* = 10 *ps*.

#### Setup: Other information

The laser pulses where focused on a single QD by a microscope objective (*NA* = 0.75). All presented measurements where performed in a cryogenic set-up at *T* = 4.2 K.

### Theoretical model

The optical Bloch equations for the V-type three-level-system with dephasing were taken from^22^ and solved by a 4^th^ order Runge-Kutta algorithm. We assumed the population decay rates to be equal for both fine structure transitions |0> to |X> and |0> to |Y>. For direct comparisons to the experimental data we considered the measured dephasing time T_2_ = 85 ps. The direct transition rate between the fine structure split states |X> and |Y> was set to zero in accordance with our experimental findings.

## Author Contributions

A.Z and D.M. conceived and designed the concept and experiment. A.D.W., D.R. and A.K.R. fabricated the sample. D.M. performed the experiments with support from S.G. and Y.A.L. J.F. developed the theoretical model. A.Z., J.F. and D.M. analysed the data and wrote the paper.

## Additional Information

**How to cite this article**: Mantei, D. *et al*. Robust Population Inversion by Polarization Selective Pulsed Excitation. *Sci. Rep.* **5**, 10313; doi: 10.1038/srep10313 (2015).

## Figures and Tables

**Figure 1:**
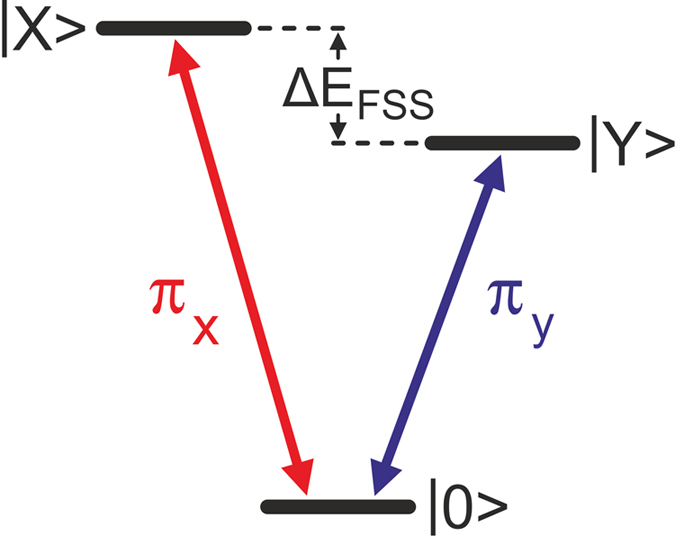
V-type three level system with a common ground state |0> and split upper states |X> and |Y>. The optical transitions π_X_ and π_Y_ can be distinguished either by polarization selection rules or by energy. A prototype for such a system is the fine structure split single exciton in a quantum dot. For a finite fine structure splitting ΔE_FSS_, the transitions are linear polarized and orthogonal with respect to each other.
